# Supplementary material for: Enhancing Accuracy of Quantum-Selected Configuration Interaction Calculations Using Multireference Perturbation Theory: Application to Aromatic Molecules
Source: ACS Omega. 2025 Aug 27;10(35):39736–50. doi: 10.1021/acsomega.5c03371 (PMC12423809; doi:10.1021/acsomega.5c03371)
Supplement: Supplementary file 1 [file ao5c03371_si_001.pdf]

Supporting Information: Enhancing Accuracy of Quantum-Selected Configuration Interaction Calculations Using Multireference Perturbation Theory: Application to Aromatic Molecules

Soichi Shirai,[*,†] Shih-Yen Tseng,[‡] Hokuto Iwakiri,[‡] Takahiro Horiba,[†] Hirotoshi Hirai,[†] and Sho Koh[*,‡]

†*Toyota Central Research and Development Laboratories, Incorporated, 41-1 Yokomichi, Nagakute, Aichi 480-1192, Japan*

‡*QunaSys Inc., Aqua Hakusan Building 9F, 1-13-7 Hakusan, Bunkyo, Tokyo 113-0001, Japan*

E-mail: shirai@mosk.tytlabs.co.jp; koh@qunasys.com

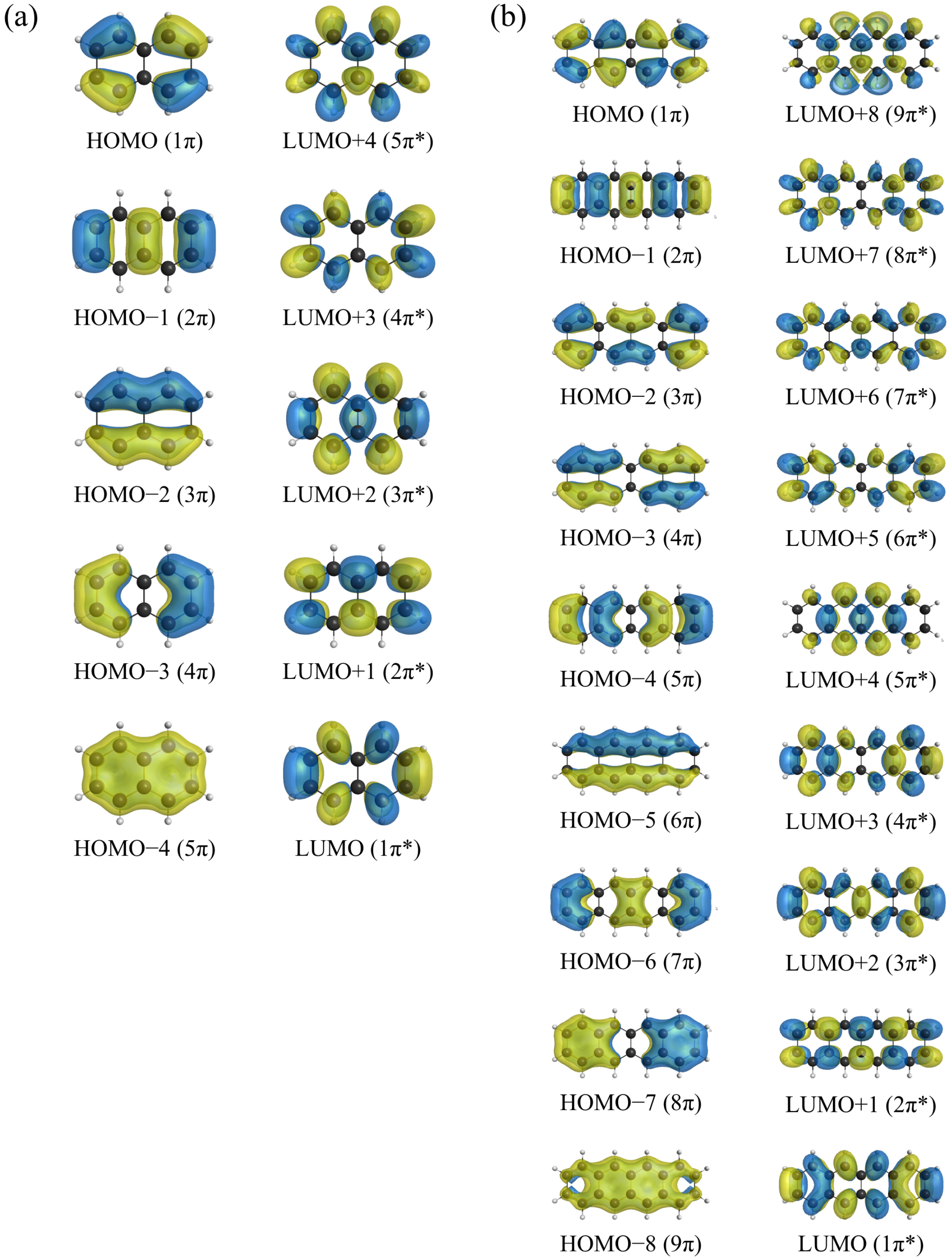

Figure S1: Valence π and π^* orbitals of (a) naphthalene and (b) tetracene. The orbitals were calculated using the Hartree-Fock method with the cc-pVDZ basis set.

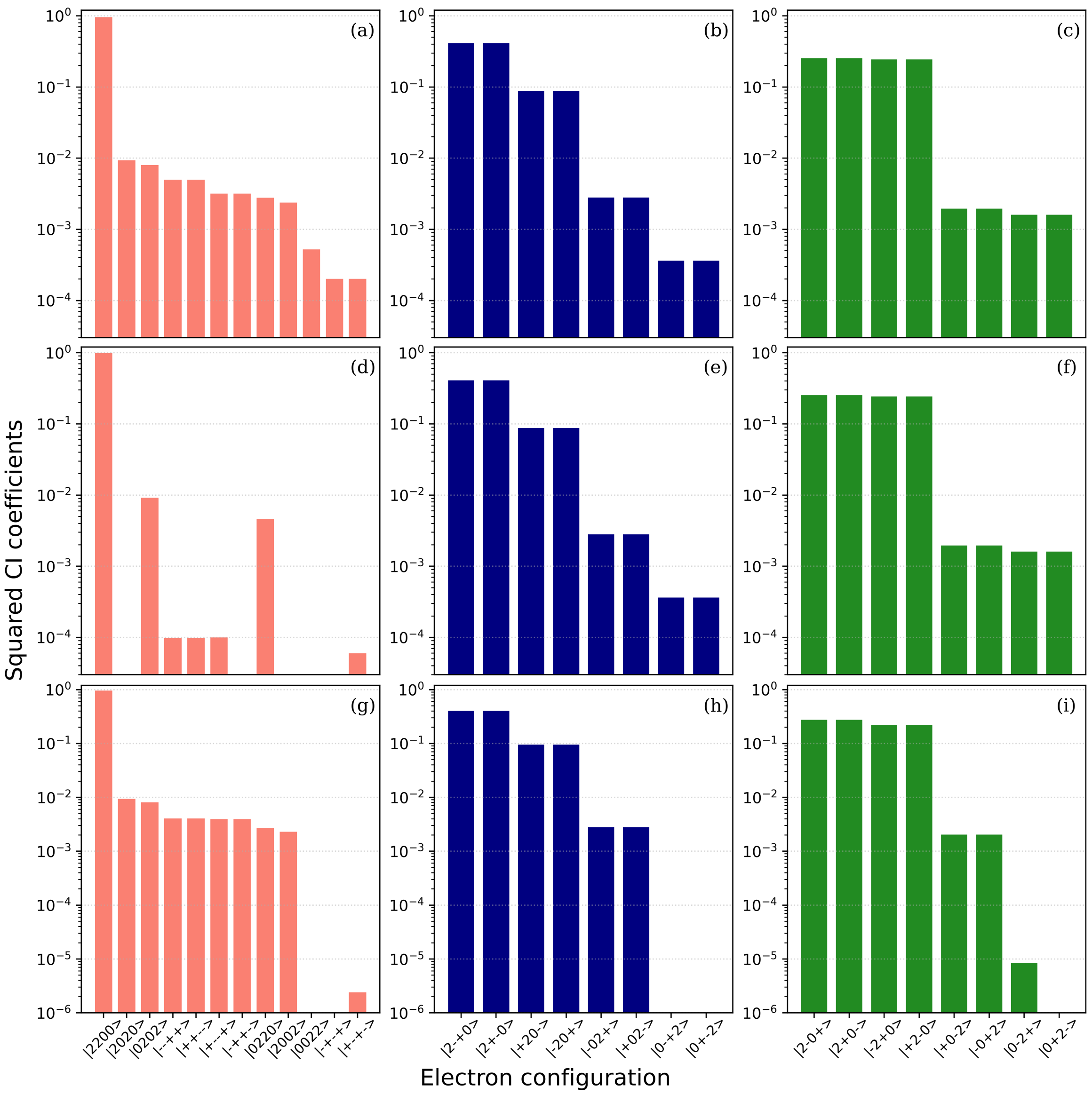

Figure S2: Squared CI coefficients of CASCI and QSCI calculations for a naphthalene molecule. The top row shows the results of CASCI for (a) the ground state, (b) the ^1L_a excited state and (c) the ^1L_b excited state. The middle row shows the results of noiseless QSCI simulation for (d) the ground state, (e) the ^1L_a excited state and (f) the ^1L_b excited state. The bottom row shows the results of QSCI using `ibmq_osaka` for (g) the ground state, (h) the ^1L_a excited state and (i) the ^1L_b excited state. The x-axis labels represent the electron configurations, whose order is chosen to align with the ones obtained from the results of CASCI calculation in the first row.

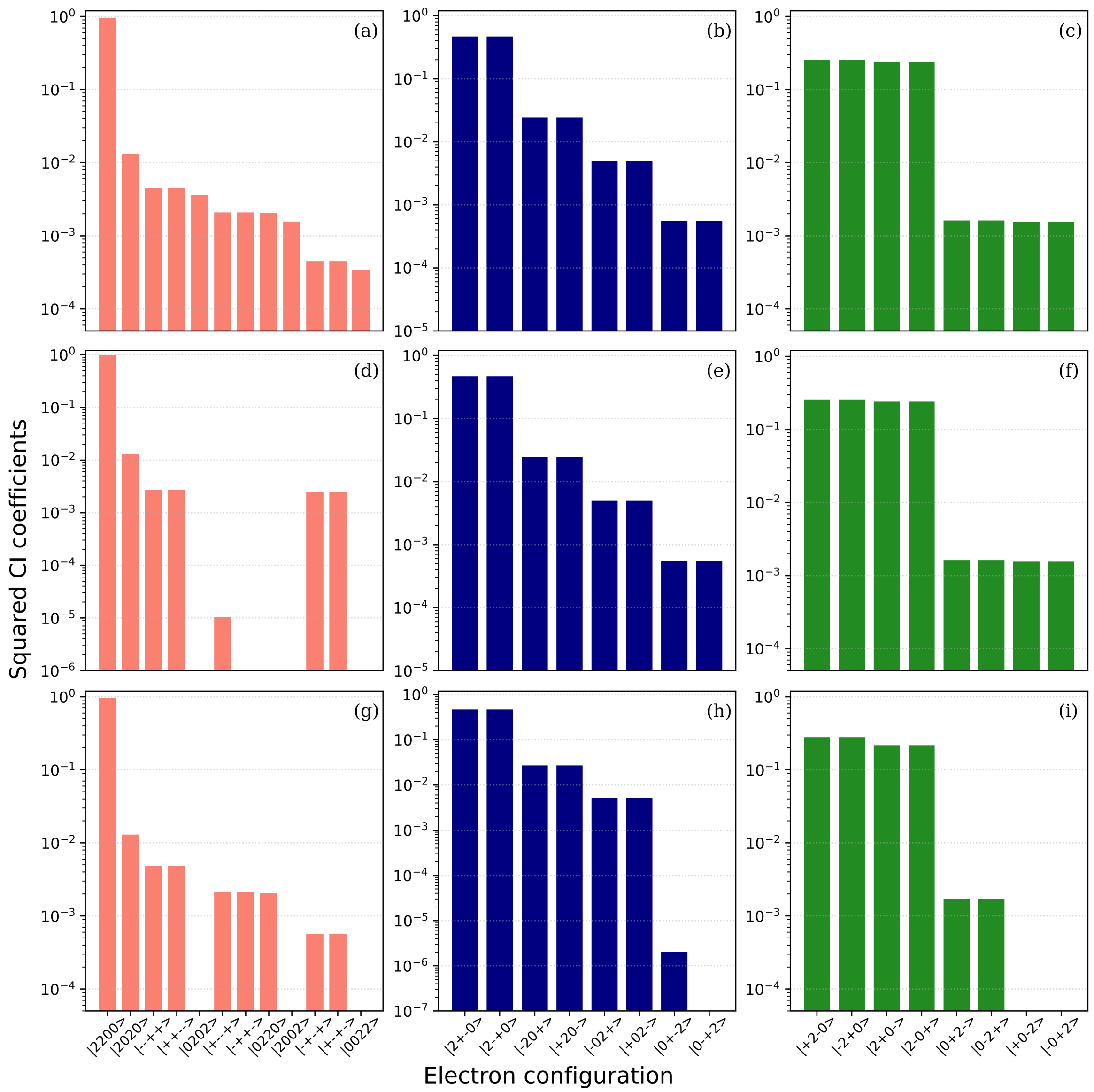

Figure S3: Squared CI coefficients of CASCI and QSCI calculations for a tetracene molecule. The top row shows the results of CASCI for (a) the ground state, (b) the $^1\mathrm{L_a}$ excited state and (c) the $^1\mathrm{L_b}$ excited state. The middle row shows the results of noiseless QSCI simulation for (d) the ground state, (e) the $^1\mathrm{L_a}$ excited state and (f) the $^1\mathrm{L_b}$ excited state. The bottom row shows the results of QSCI using `ibmq_osaka` for (g) the ground state, (h) the $^1\mathrm{L_a}$ excited state and (i) the $^1\mathrm{L_b}$ excited state. The x-axis labels represent the electron configurations, whose order is chosen to align with the ones obtained from the results of CASCI calculation in the first row.
